# Improvement in the quality of life of patients with rhododendrol‐induced leukoderma after camouflaging with dihydroxyacetone cream

**DOI:** 10.1111/1346-8138.15398

**Published:** 2020-05-18

**Authors:** Kayoko Matsunaga, Minoru Sasaki, Takao Okajima, Masahiro Miyaki, Hitoshi Sakaguchi

**Affiliations:** ^1^ Department of Integrative Medical Science for Allergic Disease Fujita Health University School of Medicine Nagoya Japan; ^2^ Biological Science Research Kao Corporation Odawara Japan; ^3^ Skincare Products Research Kao Corporation Sumida Japan; ^4^ Safety Science Research Kao Corporation Haga Japan

Rhododendrol, 4‐(4‐hydroxyphenyl)‐2‐butanol, Rhododenol^®^(Kanebo Cosmetics Inc., Tokyo, Japan) (RD) is a whitening agent, which was approved as a quasi‐drug by the Ministry of Health, Labor and Welfare of Japan in 2008.^1^ However, cosmetic products containing RD were withdrawn from the market in 2013 because some users had developed leukoderma at the sites of product application.^1^ While in more than 80% of patients RD‐induced leukoderma (RDIL) was ameliorated, it was worsened or showed no change in approximately 16%.^1^ Camouflage therapies are also often employed in cases of vitiligo to reduce the emotional stress of the patients by making the depigmented lesions inconspicuous.^2^ A self‐tanning agent containing dihydroxyacetone (DHA) is easy to apply and retains color stably for a while.^3^ As frequent applications are not needed, it is preferred in camouflaging the neck and hands, from which it may be rubbed off easily.^4^ A study has reported the usefulness of the self‐tanning lotion containing DHA in cases of RDIL with refractory lesions on the neck and hands.^5^ However, the challenges of color matching in the intricately formed depigmentation area and skin dryness after application persist. To solve these problems, we examined the usefulness of cream‐based formulations that are easy to apply and able to retain moisture in patients with RDIL.

Eight patients with refractory RDIL on the backs of the hands and/or necks, who had been treated at the Department of Dermatology, Fujita Health University Hospital, were enrolled in this prospective observational study approved by the institutional review board of Fujita Health University. All of the participants were Japanese women with an average age of 54 years (range, 44–81). We explained the study in detail to the patients, who provided written informed consent before participating.

Two types of creams, containing glycerol, diglycerol or 1,3‐butanediol as moisturizing ingredients and DHA at a concentration of 1% or 3% were used. The patients were asked to apply these creams at the site of the lesion, a maximum of twice daily for 2 months between July and September. The type of cream and frequency of application was decided by the patients themselves based on the condition of colored skin.

After the application of DHA creams for 1 month, the lesions of all patients were inconspicuous, and the camouflage lasted for up to 2 months during application. The lesions disappeared along with desquamation of the stratum corneum within 3 weeks after discontinuation of application. Typical examples are shown in Figure 1. No skin complications, such as eczema, dryness and erythema, were seen during application and up to 3 weeks after discontinuation in the assessment by an expert dermatologist. We examined the change in quality of life (QOL) as measured by using the Japanese version of Skindex‐16,^6^ which is a well‐accepted instrument to evaluate dermatology‐specific QOL.^7^ The total Skindex‐16 scores were significantly lower at 2 months than those before the commencement of application of the creams (Fig. S1a), showing considerable improvement in QOL.

**Figure 1:**
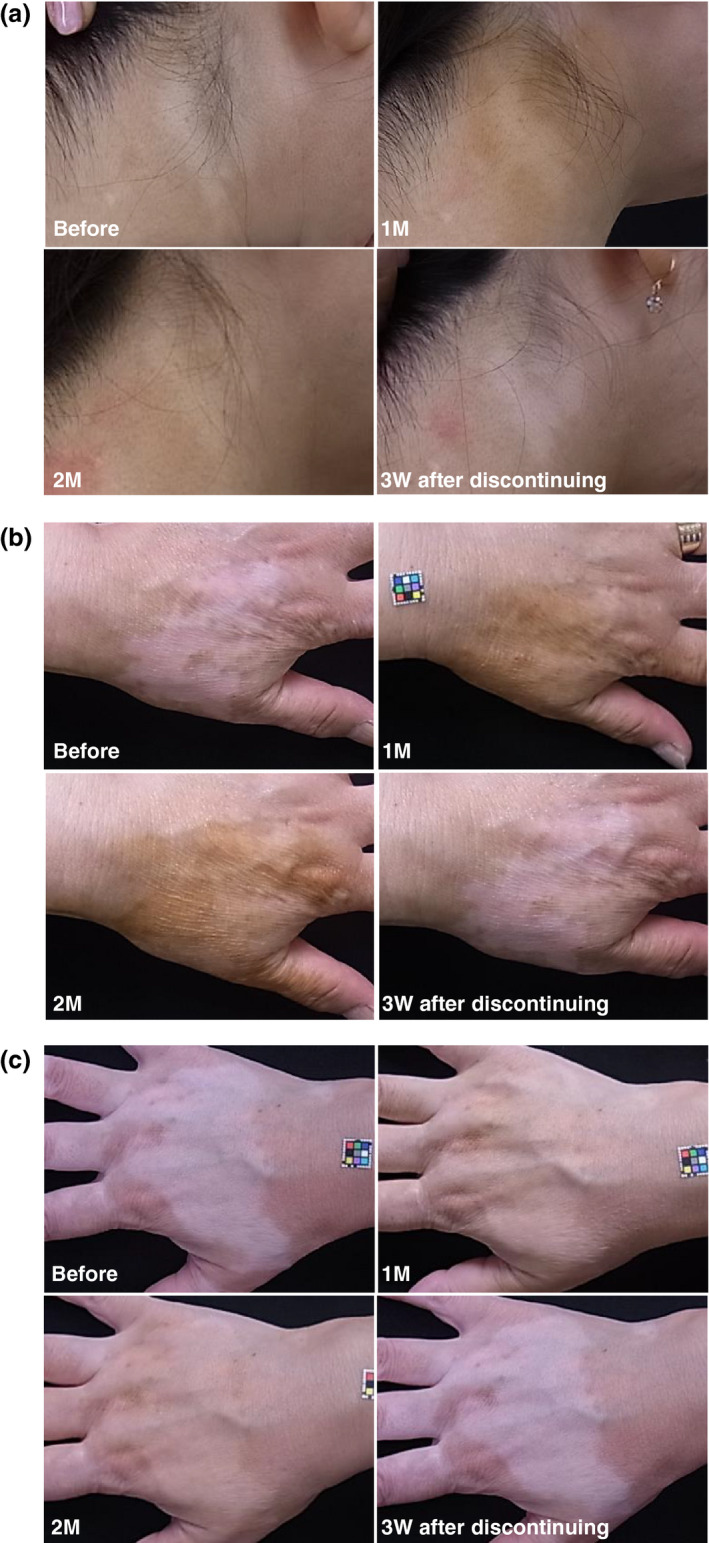
Typical skin color changes in patients with rhododendrol‐induced leukoderma. (a) Case 1: neck, before application (upper left), day 35 (upper right), day 63 (lower left) and day 14 after discontinuation of application (lower right). (b) Case 6: hand, before application (upper left), day 24 (upper right), day 59 (lower left) and day 15 after discontinuation of application (lower right). (c) Case 8: hand, before application (upper left), day 35 (upper right), day 63 (lower left) and day 14 after discontinuation of application (lower right).

Interestingly, this improvement lasted up to 3 weeks after discontinuation of the cream, suggesting that the patients were secure regarding the camouflage provided by the cream. The responses were categorized as subscales of symptoms, emotions and functions. Compared to baseline, especially, the emotions subscale score decreased significantly during application and also after discontinuation (Fig. S1b). The functioning subscale scores decreased significantly, while the symptoms subscale scores were unchanged during the entire duration of the study (Fig. S1c,d). Additionally, there were few negative answers in the self‐assessment questionnaires administered at the end of the study regarding the comfort of use, functionality of creams and usefulness (Fig. S2). Almost all patients agreed or agreed somewhat to the questions regarding the benefit of the cream and their desire to continue using.

A previous study demonstrated the usefulness of a self‐tanning lotion for the treatment of refractory RDIL; however, only half or less than half of the patients wanted to continue using it.^5^ In contrast, in this study, almost all patients showed their willingness to continue using the creams.

Meanwhile, a patient who had a complicated form of depigmentation with patchy repigmentation expressed difficulty in applying the cream to the depigmented area. Thus, we must consider the condition of depigmentation while prescribing the self‐tanning agent.

In summary, we demonstrated that DHA‐containing moisturizing creams could significantly improve the QOL of patients with RDIL. Further studies are warranted to clarify the individual‐ and site‐based differences in responding to DHA.

## CONFLICT OF INTEREST

This study was approved by the Conflict of Interest Committee of Fujita Health University (CI18‐288) and was conducted as a contract study of Kao Corporation. All of the co‐authors are employees of Kao Corporation.

## Supporting information


**Figure S1.** Improvement in quality of life (QOL) as evaluated by Skindex‐16. Changes in (a) Skindex‐16, (b) total scores, (c) emotions subscale scores, (d) functioning subscale scores and (d) symptoms subscale scores. Values are expressed as mean ± standard deviation (*n* = 8). The Wilcoxon signed‐rank test was used for statistical analysis for comparison with the baseline. *P < 0.05, **P < 0.01.

**Figure S2.** Analysis of the self‐assessment questionnaires for usefulness, ease of use and functionality. Percentage of responses (*n* = 8).
