# Supplementary material for: Improvement in the quality of life of patients with rhododendrol‐induced leukoderma after camouflaging with dihydroxyacetone cream
Source: J Dermatol. 2020 May 18;47(7):801–2. doi: 10.1111/1346-8138.15398 (PMC7383915; doi:10.1111/1346-8138.15398)
Supplement: Supplementary file 1 — Figure S1. Improvement in quality of life (QOL) as evaluated by Skindex‐16. Changes in (a) Skindex‐16, (b) total scores, (c) emotions subscale scores, (d) functioning subscale scores and (d) symptoms subscale scores. Values are expressed as mean ± standard deviation (n = 8). The Wilcoxon signed‐rank test was used for statistical analysis for comparison with the baseline. *P < 0.05, **P < 0.01. [file JDE-47-801-s001.pptx]

## Slide 1
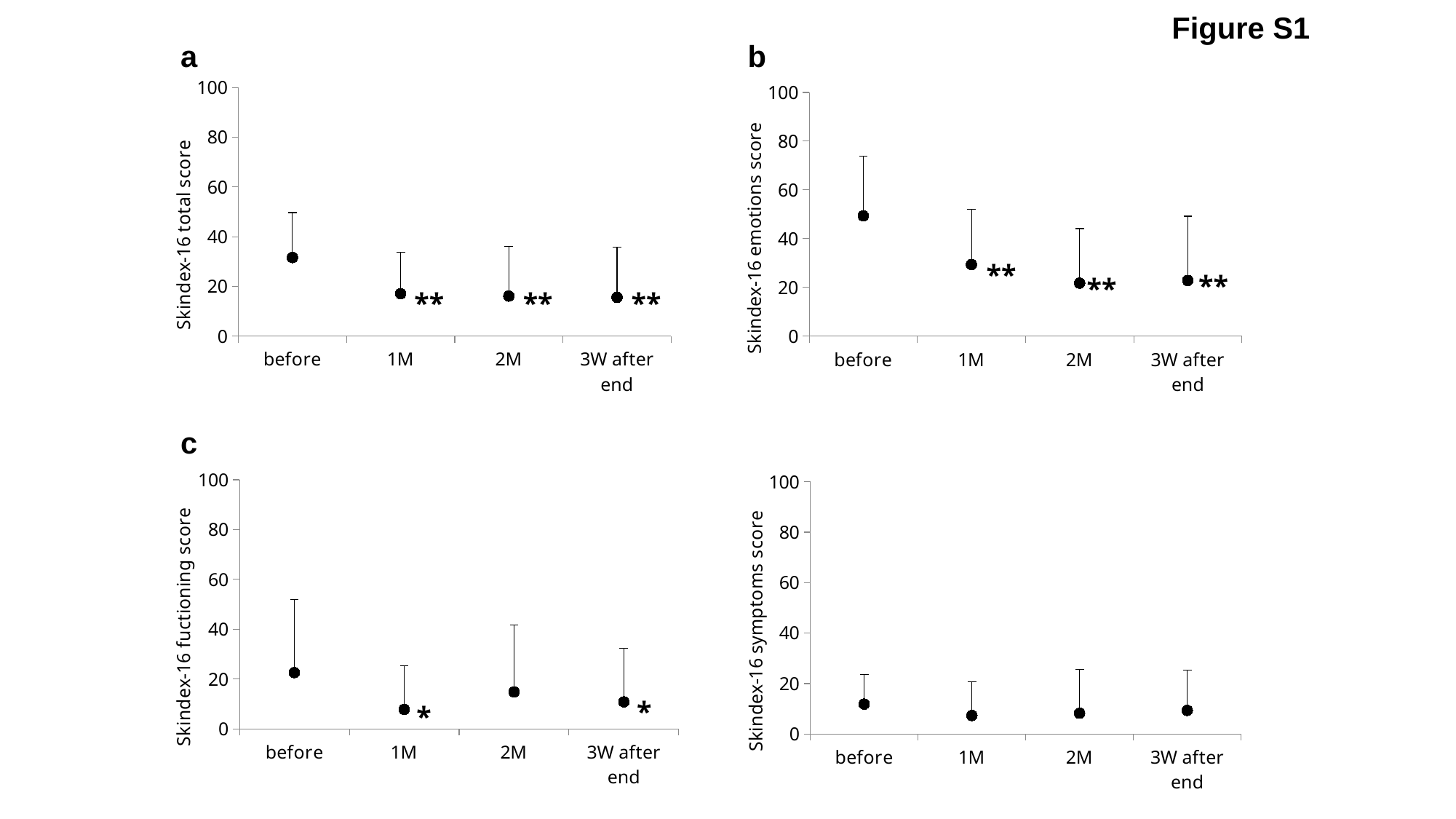

Figure S1
a
b
### Chart
| Category | 平均 |
|---|---|
| before | 31.625 |
| 1M | 17.125 |
| 2M | 16.125 |
| 3W after end | 15.625 |
### Chart
| Category | 平均 |
|---|---|
| before | 49.375 |
| 1M | 29.375 |
| 2M | 21.75 |
| 3W after end | 22.833333333333336 |**
**
**
**
**
**
c
### Chart
| Category | 平均 |
|---|---|
| before | 22.625 |
| 1M | 7.875 |
| 2M | 14.875 |
| 3W after end | 10.875 |
### Chart
| Category | 平均 |
|---|---|
| before | 11.875 |
| 1M | 7.375 |
| 2M | 8.25 |
| 3W after end | 9.375 |*
*
