# Supplementary material for: Improvement in the quality of life of patients with rhododendrol‐induced leukoderma after camouflaging with dihydroxyacetone cream
Source: J Dermatol. 2020 May 18;47(7):801–2. doi: 10.1111/1346-8138.15398 (PMC7383915; doi:10.1111/1346-8138.15398)
Supplement: Supplementary file 2 — Figure S2. Analysis of the self‐assessment questionnaires for usefulness, ease of use and functionality. Percentage of responses (n = 8). [file JDE-47-801-s002.pptx]

## Slide 1
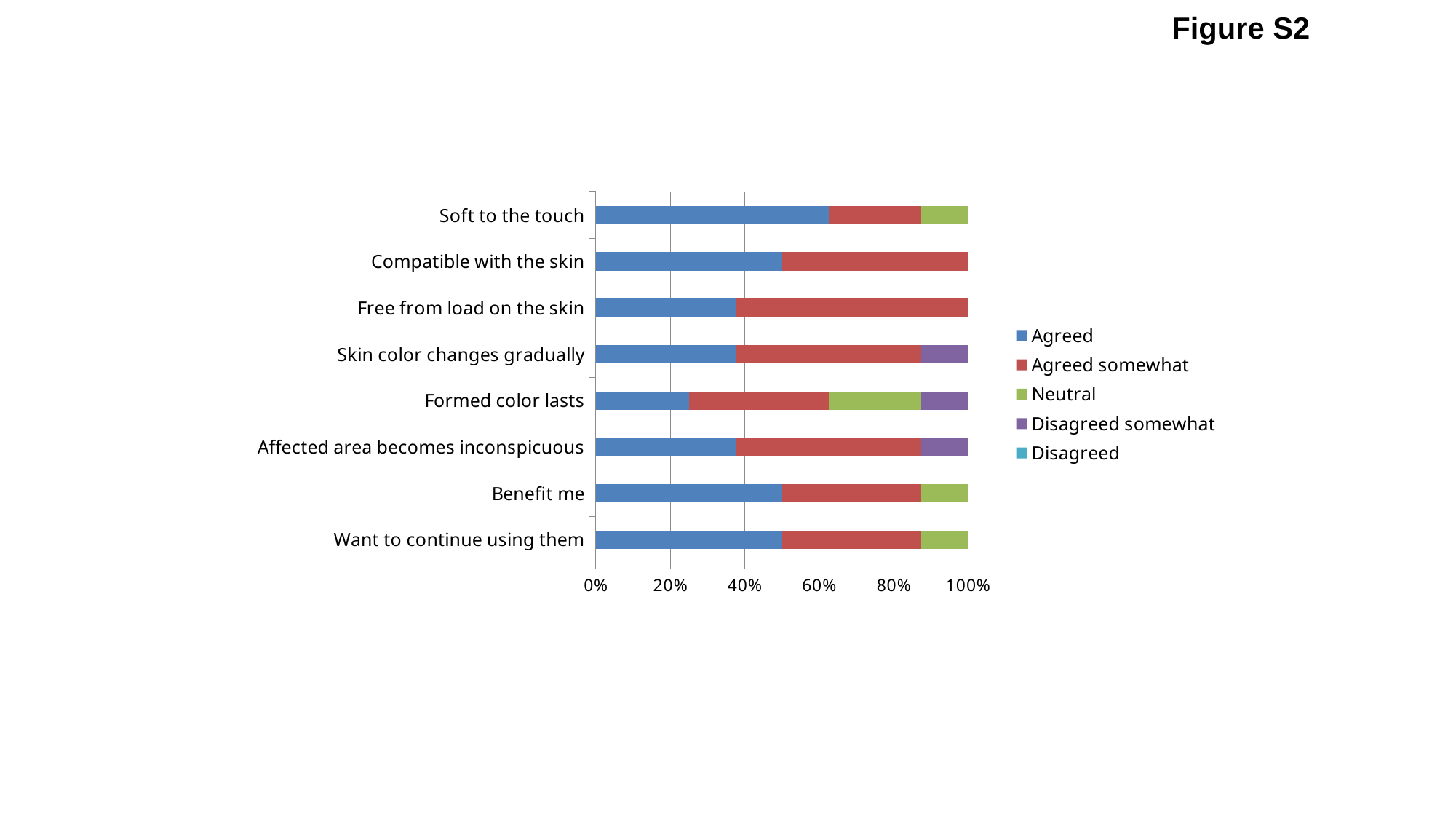

Figure S2
### Chart
| Category | Agreed | Agreed somewhat | Neutral | Disagreed somewhat | Disagreed |
|---|---|---|---|---|---|
| Want to continue using them | 4.0 | 3.0 | 1.0 | None | None |
| Benefit me | 4.0 | 3.0 | 1.0 | None | None |
| Affected area becomes inconspicuous | 3.0 | 4.0 | None | 1.0 | None |
| Formed color lasts | 2.0 | 3.0 | 2.0 | 1.0 | None |
| Skin color changes gradually | 3.0 | 4.0 | None | 1.0 | None |
| Free from load on the skin | 3.0 | 5.0 | None | None | None |
| Compatible with the skin | 4.0 | 4.0 | None | None | None |
| Soft to the touch | 5.0 | 2.0 | 1.0 | None | None |
